# Abundance, Composition and Activity of Ammonia Oxidizer and Denitrifier Communities in Metal Polluted Rice Paddies from South China

**DOI:** 10.1371/journal.pone.0102000

**Published:** 2014-07-24

**Authors:** Yuan Liu, Yongzhuo Liu, Yuanjun Ding, Jinwei Zheng, Tong Zhou, Genxing Pan, David Crowley, Lianqing Li, Jufeng Zheng, Xuhui Zhang, Xinyan Yu, Jiafang Wang

**Affiliations:** 1 Institute of Resource, Ecosystem and Environment of Agriculture, Nanjing Agricultural University, Nanjing, China; 2 Center of Ecosystem Carbon Sink and Environment Remediation, Zhejiang Agricultural and Forestry University, Linan, Hangzhou, China; 3 Department of Environment Sciences, University of California Riverside, Riverside, California, United States of America; University of Vigo, Spain

## Abstract

While microbial nitrogen transformations in soils had been known to be affected by heavy metal pollution, changes in abundance and community structure of the mediating microbial populations had been not yet well characterized in polluted rice soils. Here, by using the prevailing molecular fingerprinting and enzyme activity assays and comparisons to adjacent non-polluted soils, we examined changes in the abundance and activity of ammonia oxidizing and denitrifying communities of rice paddies in two sites with different metal accumulation situation under long-term pollution from metal mining and smelter activities. Potential nitrifying activity was significantly reduced in polluted paddies in both sites while potential denitrifying activity reduced only in the soils with high Cu accumulation up to 1300 mg kg^−1^. Copy numbers of *amoA* (AOA and AOB genes) were lower in both polluted paddies, following the trend with the enzyme assays, whereas that of *nirK* was not significantly affected. Analysis of the DGGE profiles revealed a shift in the community structure of AOA, and to a lesser extent, differences in the community structure of AOB and denitrifier between soils from the two sites with different pollution intensity and metal composition. All of the retrieved AOB sequences belonged to the genus *Nitrosospira*, among which species Cluster 4 appeared more sensitive to metal pollution. In contrast, *nirK* genes were widely distributed among different bacterial genera that were represented differentially between the polluted and unpolluted paddies. This could suggest either a possible non-specific target of the primers conventionally used in soil study or complex interactions between soil properties and metal contents on the observed community and activity changes, and thus on the N transformation in the polluted rice soils.

## Introduction

Nitrogen fertilizers are essential for rice production, but their overuse had also caused serious problems from runoff of nitrate and contributes to climate change from production of the greenhouse gas, nitrous oxide [1]. To addressing these problems, many of the environmental and management factors that affect biological transformations of nitrogen had been well studied to determine which soils are most prone to nitrogen losses [2], [3]. However, there had been still open questions on the effects of heavy metal pollution on biological nitrogen transformations, mainly owing to the difficulty in determining the extent to which microbial communities adapted to the presence of heavy metals over time when metals were slowly introduced by atmospheric deposition, versus their response to sudden increases in metal concentrations when heavy metals were spiked into soils at high concentrations [4], [5]. Another challenge in determining the effects of metals on nitrogen transformations had been the difficulty in relating changes in soil enzyme activities to changes in the abundance of molecular markers for specific microbial taxa and genes that could be involved in nitrogen transformations [6]. This is especially true for rice paddy soils where wet/dry cycles drive significant changes in redox that simultaneously affected metal bioavailability, microbial community structures, and rates for biological transformations of nitrogen.

Nitrous oxide (N_2_O) had been widely accepted as the most radiative greenhouse gas increasing at a year rate of approximate 0.26% per year such that this greenhouse gas had reached with a concentration of 319 10^−9^ mol mol^−1^ in global air by IPCC [7]. However, it had been known also as a product resulted from uncompleted denitrification, in which reduction of nitrite was not completed to form N_2_ in soils. Agriculture accounted for about 60% of the global total anthropogenic N_2_O emission, of which rice paddies had been considered a major contributor [8]–[10]. Total N_2_O emission from China’s rice paddies was estimated at 29.0 Gg N_2_O per year, accounting for 7–11% of annual overall greenhouse gas emission from mainland China croplands [9]. Ammonia oxidization to nitrite had been well known as the initial and rate limiting step in nitrification, being mediated by microorganisms which carry genes encoding for the enzymes AOB [11], [12] and/or AOA [13], [14]. In contrast to the nitrifier which could be comprised by a few functional taxa, denitrifying bacteria responsible for denitrification could be broadly distributed among many different taxa using nitrate as an alternate electron acceptor for respiration [15].

China had been the largest rice producing country in the world with approximately 20% of global rice production [16]. In the last decade, metal pollution had been widely reported to occur in extensive rice production areas that included the lower Yangtze River delta [17], [18], the Pearl River delta [19] and river valleys in the Jiangxi and Guangdong provinces [20]. Much attention had given to the potential health risk through food chain transfer of heavy metals and adverse effects on ecosystem health [21], [22]. Recently, there had been observed in metal polluted rice paddies a decline in microbial biomass and fungal to bacterial ratio with an increase in the metabolic quotient that could lead to changes in C cycling [23]. Many laboratory studies had shown that heavy metal contamination in soil could affect the rates of microbial-mediated biogeochemical processes [24], [25]. Soil nitrification rates had been known to be suppressed under metal pollution both in spiked soil samples in short term studies, and in polluted fields where metals typically accumulated at a slower rate [26]. In a study on surface wetland sediments [27], total denitrification activity was significantly decreased in multiple metal spiked wetland samples over a period of 7 days incubation with Cd, Cu or Zn ≥500 mg kg^−1^, with Cd being the strongest inhibitor. Whereas, heavy metal pollution could exert effects on ammonia oxidizers [24], [25], [28], [29] and denitrifying bacteria in field soils [30], [31].

However, changes in composition and diversity of N-transforming microbial communities with metal pollution had not yet been studied in polluted rice fields. Among these, the potential effect of metal pollution on nitrification and denitrification processes in the rice fields should require primitive study for addressing changes in N cycling in polluted lands. Thus, understanding N loss via soil-air flux of N_2_O emission and projecting future N_2_O emissions from rice soils would depend on a better understanding of how nitrification and denitrification could contribute to the soil-atmosphere N_2_O flux in rice agriculture and how these processes could be affected by heavy metals. In addition, nitrifier and denitrifier could also have different responses to stress of heavy metal pollution in soils owing to their different resource requirements, different metal contamination would exert different effects on the changes in the microbial communities and their biological functions on mediating N transformations in polluted rice fields.

Here we hypothesize that heavy metal contamination could have impact in the abundance, composition and activity of nitrifying and denitrifying communities, which could differ in soils with different soil properties as well as with different metal composition. An experiment was conducted to compare the community composition of nitrifier and denitrifier using two long-term metal polluted soils, which were compared to those in adjacent unpolluted fields at each location. We further studied the possible linkage between gene copy numbers that could serve as molecular markers for nitrogen transformation processes and process rates determined by direct enzyme assays measuring the potential activity of the nitrifying and denitrifying communities.

### Ethics statement

The contaminated soils that we collected samples were under rice paddy used for rice production. No specific permission required nor any endangered or protected species involved. The polluted paddies both had a 30 year history of metal pollution due to discharge of mining waste water.

## Materials and Methods

### Site description

Two locations with soil pollution were selected for this study. Location DX (29°04′N, 117°43′E, Dexing County, Jiangxi) was situated in a copper mining area. The second location, designated DBS (24°26′N, 113°49′E, Wenyuan Municipality, Guangdong Province), was close to a multiple metal mine of zinc, cadmium, lead, and copper. The polluted paddy of at DX site was 1.5 km in distance from the top-hill ore mining and 1 km in distance to an adjacent Cu smelter, being affected by waste water discharge and atmospheric deposition. Whereas, the polluted paddy at DBS was polluted with irrigation by river water discharged from an upstream multiple metal mining ore and the associated smelter 3.5 km in distance. The mining and smelter activity had been taken place since late 1960’s in both sites [23]. In each location, unpolluted rice paddies were selected that had no distinct access to polluted irrigation water. Soil samples from the polluted and non-polluted rice paddies at each location were designated as polluted (PS) and background soil (BGS) respectively. The climate at both locations are characterized by a subtropical monsoon climate with a mean annual temperature from 18°C to 25°C, and mean annual rainfall from 1200 mm to 1450 mm. In agriculture, double rice cropping has been traditionally practiced in the rice paddies in the area where these sites situated.

### Soil sampling

Soil sampling was conducted at each location before rice planting in spring of 2009. Three composite topsoil samples at a depth of 0–15 cm were randomly collected respectively in 3 subplots from both PS and BGS fields. Each composite sample consisted of 5 sub-samples that were collected following a “*Z*” shaped sampling pattern with a distance of ∼5 m in each subplot. The composite samples were mixed thoroughly and kept on ice until they were transported to the laboratory within two days after sampling. The samples were processed to remove gravels and plant detritus if any, and passed through a 2 mm sieve. Each composite sample was divided into three portions, one of which was used for DNA extraction. A second portion was stored at 4°C for measuring potential nitrifying and denitrifying activities. The third portion was air-dried at room temperature for analysis of soil chemical and physical properties as described below.

### Analysis of soil properties and metal contents

Measurements of the basic properties and metal contents of the samples were conducted following the protocols described by Lu [32]. Briefly, soil pH was measured with a glass electrode using a 1/2.5 soil/water ratio. Soil textural class and particle size fractions were determined with a hydrometer method after dispersion with 0.5 mol L^−1 ^NaOH solution. Soil organic carbon (SOC) was measured using wet digestion and oxidation with potassium dichromate. Total nitrogen was analyzed using the Kjeldahl method. For total heavy metal content determination, the samples were digested with a solution of HF/HClO_4_/HNO_3_ (10/2.5/2.5, v/v/v) and then extracted with 1 mol L^−1^ HCl. Content of Cd was determined with graphite furnace atomic absorption spectrometry (GFAAS, SpectrAA220Z, Varian, USA) while those of Pb, Cu and Zn with flame atomic adsorption spectrophotometry (FAAS, TAS-986, China). Soil physic-chemical properties and metal contents determined are presented in Tables 1 and 2, respectively.

**Table 1:** Soil physicochemical properties of the studied soil samples.

Site	Plot	pH (H_2_O)	SOC (g kg^−1^)	TN (g kg^−1^)	Clay (%)	Silt (%)	Sand (%)
DX	BGS	4.87±0.05	22.79±1.58	1.58±0.05	21.0	31.2	47.8
	PS	4.10±0.06	22.25±0.35	1.96±0.07	27.0	32.2	40.8
DBS	BGS	5.58±0.13	15.23±0.60	1.03±0.03	22.2	23.2	54.6
	PS	5.45±0.16	19.11±0.68	1.49±0.08	27.0	28.8	44.2

BGS: Background soil; PG: Polluted soil. SOC: soil organic carbon; TN: total nitrogen.

**Table 2:** Total heavy metal contents and Nemerow pollution index (Means ± S.D.) of the studied soils.

Sample	Cd (mg kg^−1^)	Pb (mg kg^−1^)	Cu (mg kg^−1^)	Zn (mg kg^−1^)	Nemerow index	Pollution intensity
DX-B	0.48±0.14b	58.95±0.90b	640.19±2.98b	94.57±17.21b	9.45±0.03b 19.74±1.86a	/
DX-P	1.55±0.14a	95.17±7.07a	1333.68±129.72a	163.90±15.41a		3.23±0.15B
DBS-B	0.29±0.00b	33.37±2.08b	21.87±1.02b	70.40±1.5b	0.76±0.001b 4.04±0.56a	/
DBS-P	1.49±0.24a	133.27±6.67a	224.83±5.68a	248.48±5.87a		5.32±0.23A

Different lowercase characters indicate significant different (*p*<0.05) between polluted soils (PS) and background soils (BGS) in a single site. Different capital letters for pollution intensity indicate significant different (*p*<0.05) between the two sites.

The Nemerow pollution index [33] was used to evaluate the overall extent of heavy metal pollution. It was calculated using the following equation:
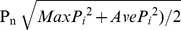
(1)


Where, P_n_ was the Nemerow pollution index value, and calculated as the sum of n metal elements analyzed for a soil sample; *P_i_* was a single pollution intensity index of *i*th metal element with its measured concentration (*C_i_*) divided by the guideline standard of environmental quality (*R*S_i_). *MaxP_i_* and *AveP_i_* were the maximum and average pollution intensity respectively of the analyzed metals in a given soil. A relative metal accumulation degree using content of a single element in PS over in BGS was calculated and then averaged as the overall pollution intensity in a single site. All these calculated values are given in Table 2.

### DNA extraction and real-time PCR assay

Total DNA was extracted from 0.25 g fresh soil with a PowerSoil DNA Isolation Kit (Mo Bio Laboratories Inc., CA) according to the manufacturer’s protocol. The primers and thermal cycling procedures are listed in Table S1. The primer sets of *nirK*876 and *nirK*1040 were used in this study as they had been considered representing typical denitrifying bacterial community in multiple samples [36]. Each reaction was performed in a 25 µl reaction volume containing 15 ng of DNA, 1 µl of 10 µM of each primer and 12.5 µl of SYBR premix EX Taq TM (Takara Shuzo, Shinga, Japan). The amplified PCR products of *amoA (AOB and AOA)* and *nirK* genes were purified using PCR solution purification kit (Takara), ligated into pEASY-T3 cloning vector (Promega, Madison, WI) and cloned into *Escherichia coli* DH5α. Clones containing correct inserts were chosen as the standards for real-time PCR (qPCR). High amplification efficiencies of AOB-*amoA* (101%), AOA-*amoA (97%) and nirK* (102%) were obtained for gene quantification, with r^2^ values of 0.996, 0.997 and 0.991 respectively.

### PCR-DGGE of ammonia oxidizing and denitrifying communities

Profiles of the ammonia oxidizing and denitrifying communities were generated by PCR-DGGE using the primer sets *amoA*-1F-GC and *amoA*-2R, which had been considered specific for the AOB [34], Arch-*amoA*F-GC and Arch-*amoA*R which could be specific for AOA [35] and the *nirK*876-GC and *nirK*1040 set for the denitrifying bacterial communities [36]. The GC clamp described by Muyzer and Smalla [37] was incorporated into the 5′ end of primers. PCR reactions were performed with reaction mixtures that contained 12.5 µl Go TaqH Green Master Mix (Promega, Madison, WI), 1 µl of 10 µM of each primer, and 1 µl of DNA template. For DGGE analysis, PCR products were separated on an 8% (w/v) polyacrylamide gel of acrylamide/bisacrylamide (37.5/1, v/v) containing denaturing gradients of 45–60% for AOB, 25–50% for AOA and 50–65% for *nirK*. DGGE was performed in 1× TAE buffer at 60°C, 200 V for 5 min, then 140 V for 500 min. Gels were silver stained [38] and scanned using a gel document system (Bio-Rad, USA).

### Sequencing and phylogenetic analysis

Prominent bands from the DGGE gels for the AOB and denitrifying communities were numbered and excised from the gels for sequence analysis. The retrieved sequences were compared with GenBank data base sequences using BLAST (Basic Local Alignment Search Tool) (http://www.ncbi.nlm.nih/gov/blast/) to search for best matches. Sequences of the DGGE bands had been deposited in GenBank under the accession numbers JF264803 to JF264813 (AOB) and JF264827 to JF264835 (*nirK*).

### Measurement of potential nitrification and denitrification activities

Potential nitrification activity (PNA) was measured using the short-term nitrification assay described by Schmidt and Belser [39]. Briefly, 20 g moist soil was added to a 250 mL cotton-stopped flask with 100 ml of 0.5 mM phosphate buffer (pH 7.2), 0.5 mM (NH_4_)_2_SO_4_ and 10 mM KClO_3_ to stop further oxidation of NO_2_^–^. Triplicate flasks for each treatment were incubated for 24 h on a shaker at 175 rpm at 25°C. Net rate of NO_2_^–^ production was calculated based on the linear correlation of NO_2_^−^ concentration versus time.

Potential denitrification activity (PDA) assay was analyzed by the acetylene (C_2_H_2_) inhibition method following the procedure described by Tiedje et al. [40]. N_2_O was measured using a gas chromatograph (Agilent 7890D, Santa Clara, CA, USA). Assays were conducted by addition of 20 g moist soil at 60% WHC to 250 mL glass bottles, in soil slurries produced by adding 20 mL of a substrate solution containing 1 mM glucose and 1 mM KNO_3_. The linear rates of N_2_O production over time were observed within 6 h of initiating the incubations.

### Data processing and statistical analysis

The data were processed with Microsoft Excel 2010 and expressed in means plus/minus standard deviations. A paired t-test was used to evaluated differences between the polluted (PS) and background (BGS) samples from a single site with a significance defined at *p*<0.05. Digitized DGGE images were analyzed with Quantity One image analysis software (Version 4.0, Bio-Rad, USA). This software identified the band with the same position in the different lanes of the gel and also measures the intensity of identified bands. Cluster analysis and principal component analysis (PCA) of the DGGE profiles were performed to elucidate the microbial community structures based on relative band intensity and positions using the Minitab v.15 software. Redundancy analysis (RDA) was used to evaluate the relationship among DGGE fingerprints and soil properties (pH, SOC, TN, Nemerow index; total concentration of Cd, Pb, Cu and Zn) using the Canoco 4.5 software.

## Results

### Soil physicochemical properties and heavy metal pollution

As showed in Table 1, there were generally hardly differences in the basic properties between PS and BGS in a single site. For example, soil pH of the background soil at the DBS site (pH 5.58) was higher than at DX site (pH 4.87), without remarkable difference between PS and BGS in a single site. SOC and TN ranged from 15.23 g kg^−1^ to 22.79 g kg^−1^ and from 1.03 g kg^−1^ to 1.96 g kg^−1^ for the two sites, respectively. Here, N was generally higher in polluted soil than the background soil for less N consumed by rice with reduced production under metal pollution in both sites. However, C/N ratio seemed similar between the two sites both of polluted soils (14.4 and 14.8 respectively for DX and DBS) and of background soils (11.4 and 11.8 respectively for DX and DBS). As listed in Table 2, there were consistent differences in contents of total Cd, Pb, Cu and Zn between PS and BGS in a single site though the contents of a single heavy metal element varied with sites. While the total Cu content in PS was approximately 6 times higher from DX site than from DBS site, though a high Cu content up to 640 mg kg^−1^ owing to the lithological background [23]. As calculated based on environmental quality guideline value of China, Nemerow pollution index value was 4.0 for DBS and 20 for DX. While Cd, Pb and Zn contents of PS were similar between the two sites, the overall metal pollution intensity in PS estimated of the contents of all individual elements in PS over the BGS was significantly higher in DBS site than in DX site.

### Abundances of ammonia oxidizer (AOB and AOA) and denitrifier (*nirK*)

The relative abundances of *amoA* and *nirK* gene copy numbers were assessed by *q*PCR (Table 3). The results showed that copy numbers of AOA *amoA* gene of the two soils ranged from 1.1×10^8^ to 4.6×10^8^ g^−1^ soil, and approximately 10–100 fold greater than those of AOB *amoA* genes, which ranged from 6.4×10^6^ to 5.8×10^7^ g^−1^ soil. Compared to background soil, copy numbers of AOB *amoA* gene were apparently reduced in PS by 19% at DX but 80% at DBS, corresponding to their metal pollution intensity difference. However, copy numbers of the AOA *amoA* gene were significantly reduced in polluted soil only at DX. Accordingly, the ratio of AOA to AOB in PS decreased at DX site but increased at DBS site while AOA genes were present in higher copy numbers than AOB genes at both sites, with ratios of AOA to AOB ranging from 2.5 to 59 across all samples.

**Table 3:** Ammonia oxidizer and denitrifier gene copy numbers and the relative ratios (Means ± S.D.) of the soils studied.

Sample	AOB (×10^7^)	AOA (×10^7^)	*nirK* (×10^8^)	Ratio of AOA to AOB	Ratio of *nirK* to *amoA*
DX-B	0.79±0.07a	46.40±4.16a	6.25±1.86a	59.02±5.84a	1.32±0.35b
DX-P	0.64±0.05b	10.80±0.31b	8.33±0.25a	16.97±0.61b	7.92±0.20a
DBS-B	5.75±0.52a	14.80±5.78a	2.32±0.48a	2.55±0.89a	1.13±0.03a
DBS-P	1.13±0.06b	12.40±8.41a	3.38±0.68a	10.86±4.36a	3.34±1.03a

Different lowercase characters indicate significant different (*p*<0.05) between polluted soils (PS) and background soils (BGS) in a single site.

Copy numbers of the *nirK* gene ranged from 2.32×10^8^ to 8.33×10^8^ g^−1^ soil in the studied paddies, while there was no significant change between PS and BGS in a single site. The ratios of *nirK*: *amoA* (sum of AOA and AOB gene copy numbers) ranged from 1.13 to 7.92 across all of the soil samples. Compare to GBSs, the *nirK*: *amoA* ratios increased in polluted soils from both locations (Table 3).

### Community structures of ammonia oxidizer (*amoA*) and denitrifier (*nirK*)

**Figure 1:**
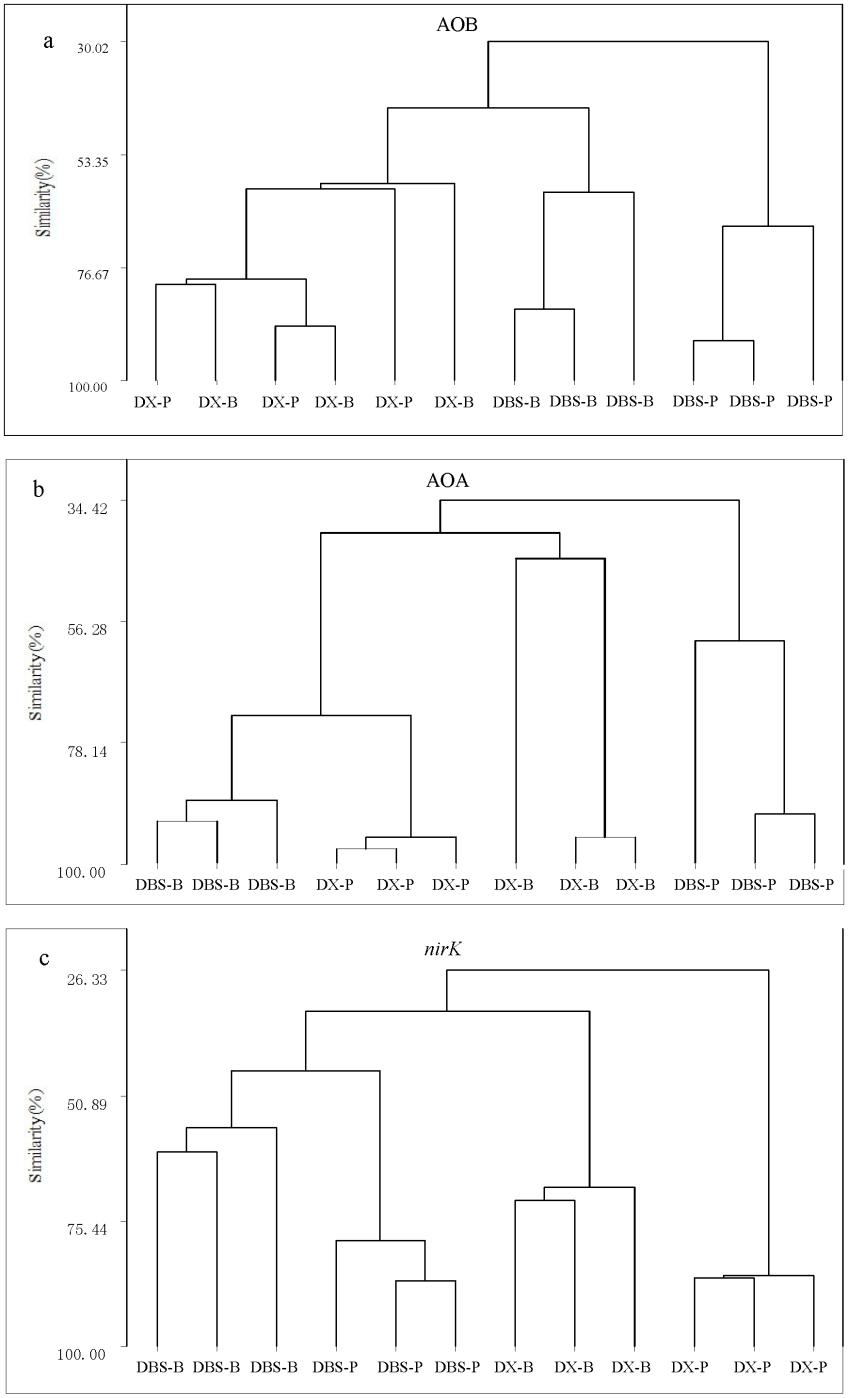
The cluster analysis of DGGE profiles of AOB (a), AOA (b) and *nirK* (c) gene fragments from the soil samples at the two sites. DX-B and DBS-B, background soil from site DX and DBS; DX-P and DBS-P, polluted soil from site DX and DBS. Euclidean distances were calculated from relative positions and intensities of bands, and the samples were clustered using Pearson’s product-moment coefficient and a UPGMA (Unweighted Pair Group Methods with Arithmetic mean) algorithm.

Cluster analysis and principal component analysis (PCA) were used to group sampled soils based on similarity in relative band intensity and position of the DGGE profiles. PCA of DGGE profiles of AOB and AOA at the two sites yielded good summaries of data, as 86% (AOB) and 76% (AOA) of the total variability was explained by the first two components (Fig. S1). The AOB community did not show clear shifts between PS and BGS soils at DX site, but was separated between of PS and BGS of DBS site on the basis of PC2. Cluster analysis of AOB community from samples with or without heavy metal pollution revealed similarity of 59% at the DX site and 30% at the DBS site (Fig. 1a). In contrast, the AOA community profiles showed well separation between PS and BGS soils at each site, and separated on the basis of PC1 for DBS samples and on the basis of PC2 for DX samples (Fig. S1b). Cluster analysis clearly showed that AOA community under PS was distinguishable from BGS soils in both sites with an intragroup similarity of 40% for the DX site and 34% for the DBS site (Fig. 1b). Both PCA and cluster analysis of DGGE profiles of *nirK* gene revealed distinct communities between PS and BGS soils at the DX site with 26% similarity; but no clear differences in *nirK* community structure at the DBS site with 48% similarity.

### Phylogenetic analysis

**Figure 2:**
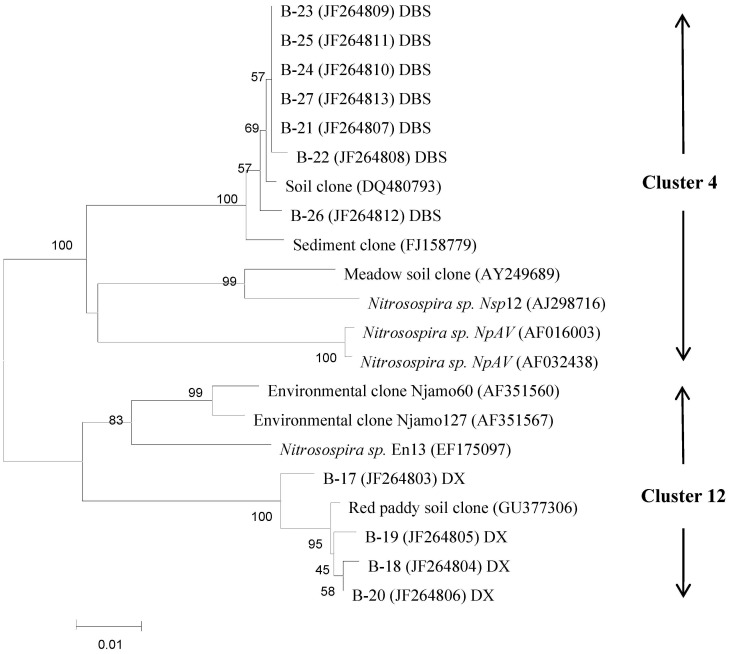
Neighbor-joining phylogenetic tree of AOB *amoA* sequences retrieved from the numbered DGGE bands of Fig. S2(A). Designation of the clones in the dashed line frames includes the following information: excised DGGE band number, accession number in the parentheses, followed by the sampling plot the clone retrieved from. Bootstrap values (>40%) with 1000 replicates are indicated at branch points. Scale bar indicates 1 change per 100 nucleotide positions.

**Figure 3:**
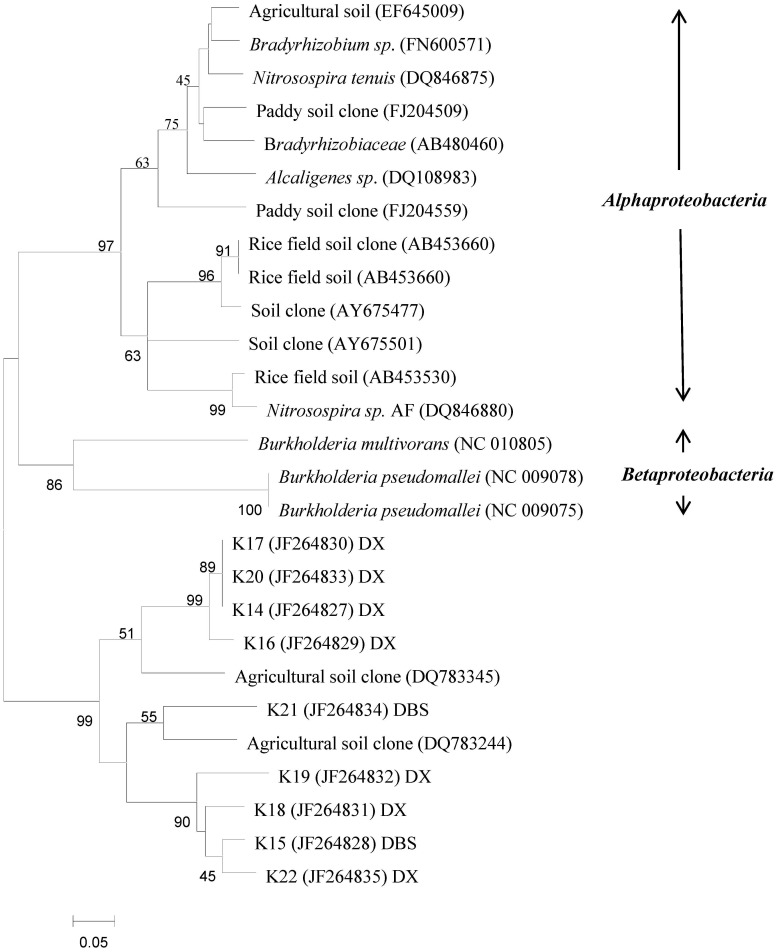
Neighbor-joining phylogenetic tree of *nirK* sequences retrieved from the numbered DGGE bands of Fig. S2(C). Designation of the clones in the dashed line frames includes the following information: excised DGGE band number, accession number in the parentheses, followed by the sampling plot the clone retrieved from. Bootstrap values (>40%) with 1000 replicates are indicated at branch points. Scale bar indicates 1 change per 100 nucleotide positions.

Selected DNA bands from the DGGE profiles were sequenced to identify the predominant taxa associated with these bands and the effects of metal pollution on specific taxa (Fig. S2). Some bands were present in the profiles from both BGS and PSs, but their intensity varied between locations. The results showed that all of the gene sequences represented in the DGGE profile of AOB *amoA* gene were associated with the genus *Nitrosospira*. The DGGE profile of AOB revealed several bands (B17, B18, B19 and B20) that were present in both PS and BGS from the DX site, and that had the same relative positions as bands present in BGS from the DBS site. However, sequences identified from the BGS at the DBS site belonged to Cluster 4, whereas bands B17–B20 from the DX site were affiliated with Cluster 12 (Fig. 2). None of the sequences of *nirK* from the different soils were affiliated with either *α-proteobacteria* or *β-proteobacteria* (Fig. 3). Whereas, most of the clones in the PS at the DBS site represented different taxa from those in BGS, sequences representing taxa from the DX site were similar for both BGS and PS.

### Soil potential nitrifying and denitrifying activities

**Figure 4:**
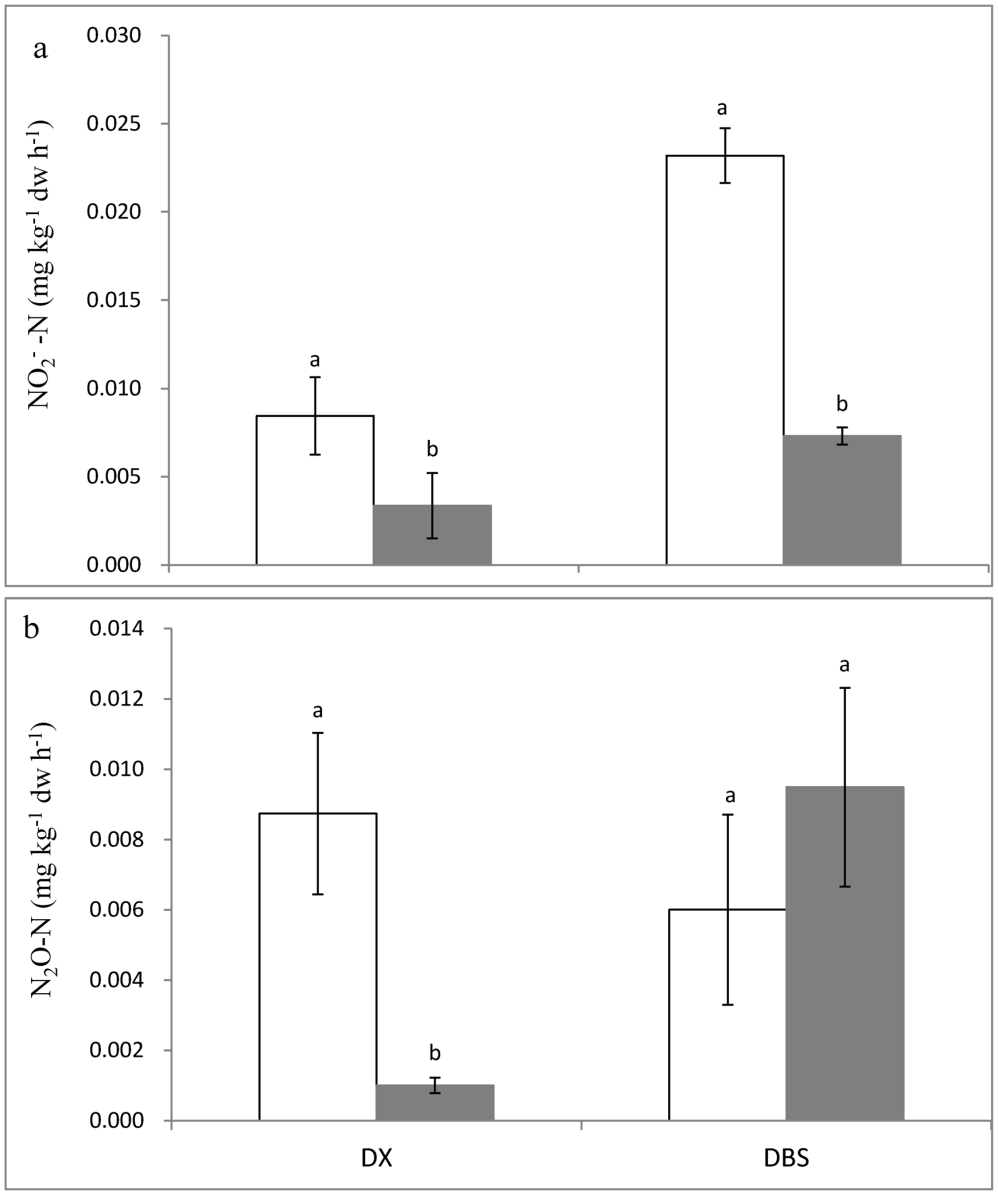
The potential nitrifying activity (a) and denitrifying activity (b) of the background (Blank) and polluted (Shaded) soils in DX and DBS sites. Different lowercase characters indicate significant difference (*p*<0.05) between the background and polluted soils in a single site.

Potential nitrifying and denitrifying activities and differences associated with metal pollution varied with location. Transformation of ammonia to nitrate was approximately 3-fold lower in PSs compared to BGS at both locations (Fig. 4a). BGSs from both the DX and DBS sites released 0.008 and 0.023 mg NO_2_^−^ -N kg^−1^ soil h^−1^, respectively, which compared to 0.003 mg NO_2_^−^ -N kg^−1^ soil h^−1^ at the DX site and 0.007 mg NO_2_^−^ -N kg^−1^ soil h^−1^ at the DBS site in BSs. An 8-fold decrease in denitrification rates was found of PS (0.009 mg N_2_O -N kg^−1^ soil h^−1^) over that of BGS (0.001 mg N_2_O -N kg^−1^ soil h^−1^) at the DX site despite of no significant change between PS and BGS at the DX site. (Fig. 4b).

### Correlation of microbial community structure with soil properties

**Figure 5:**
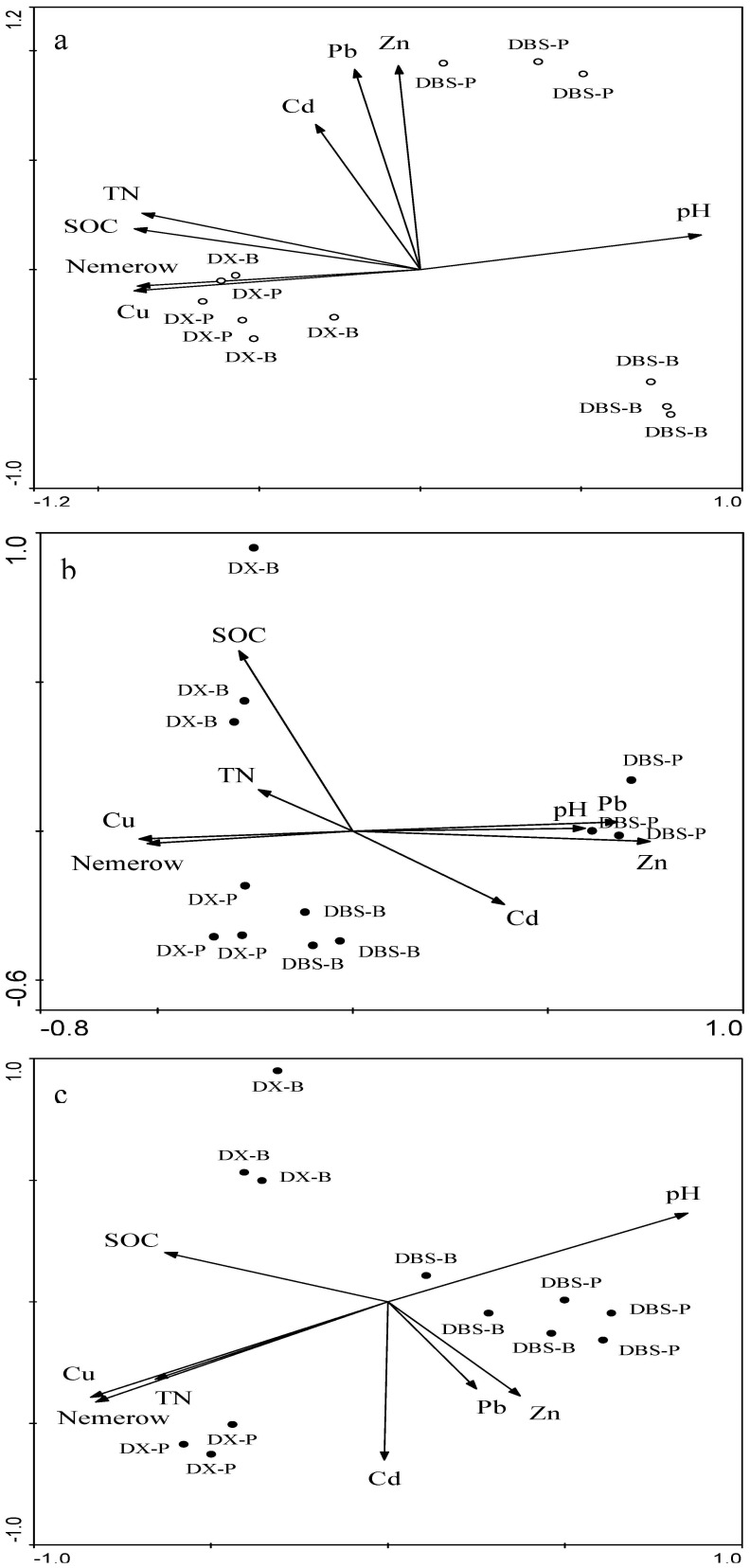
Redundancy analysis ordination plots of relationships between DGGE patterns of AOB (a), AOA (b) and *nirK* (c) gene and soil properties from the soil samples at the two sites. Soil properties included nemerow toxicity index, soil pH, SOC, TN, total concentration of Cd, Pb, Cu and Zn. DX-B and DBS-B, background soil from site DX and DBS; DX-P and DBS-P, polluted soil from site DX and DBS.

Using redundancy analysis, we identified the factors which can best explained soil microbial community structure at each site (Fig. 5). In the redundancy analysis biplot, axis 1 and axis 2 values explained 80.2% and 79.7% of the variability, respectively, in AOB profiles and AOA profiles. Soil properties most important in explained AOB community composition were soil pH, SOC, TN, Nemerow index and Cu concentration, which were strongly related to the first axis, and soil pH, Nemerow index and Cu, Pb, Zn concentration, which were most important soil factors in determining AOA community composition. In the redundancy analysis biplot of *nirK* gene fragments, axis 1 explained 45.6% and axis 2 explained 29.3% of the variability. Soil pH, TN, Nemerow index and Cu concentration were strongly related to the first axis, and Cd concentration was strongly related to the second axis (Fig. 5c).

## Discussion

### Changes in ammonia oxidizer and denitrifier populations with metal pollution

Our results showed a strong impact of metal pollutants on ammonia oxidizer with the degree varying with the metal pollution situation in the two different locations. Copy numbers of *amoA* genes for eubacteria (10^6^ to 10^7^ per g dry soil) and archaea (10^8^ per g dry soil) in the BGSs were both within the ranges that had been reported in previous studies of agricultural soils. In contrast to genes encoding enzymes for ammonia oxidation, the abundances of *nirK* genes representing the denitrifying bacteria seemed not affected by pollution in a single site. The *nirK* gene abundances ranged from 1.5×10^8^ to 8.3×10^8^ copies g^−1^ dry soil, which seemed within the range of those reported by Dandie et al. [41]. Decreases in copy numbers of *nirK* and *nosZ* genes were reported for estuary sediments with spiked copper, though recovered after prolonged incubation [31]. Likewise, in a study by Holtan-Hartwig et al. [42], a mixture of Cd, Cu and Zn caused a temporary reduction in N_2_O production in a sandy loam soil, which recovered within two months after spiking. Our results could be thus in general agreement with the hypothesis that denitrifier populations underwent adaptation to Cd, Pb, Cu and Zn pollution.

Sensitivity of ammonia oxidation to heavy metals had been known dependent on the specific metal or combination of metals. For example, a study by Liu et al. [29] showed no change in AOA abundance and community composition in a mercury-spiked vegetable soil. However, a work by Li et al. [28] showed a significant decrease in AOA abundance in four different soils (grassland and arable soils) with Cu amendments up to 1000 mg kg^−1^. In a spiked study by Fait et al. [43], the ammonia oxidizer community was shown more vulnerable to Cu pollution than to Ni. The strong effects of copper on AOB populations was also shown in a study by van Beelen et al. [44], who showed that microbial communities more readily adapted to Zn and Ni contamination than to contamination from Cu and Cr in a sludge-amended soil. Therefore, AOA and AOB could be both significantly affected by metal pollution, but that AOA seemed more strongly reduced in Cu-polluted soil.

In this study, microorganisms carrying genes encoding for AOA were more abundant than those expressing AOB, with a ratio of AOA to AOB gene copy numbers ranging from 2.5 to 59. Moreover, the ratio of AOA/AOB gene copy numbers was 71% lower in PS at DX site but did not significantly changed in those at DBS site (Table 3). Ruyters et al. [25] reported an increase in AOA/AOB ratios in a grassland soil exposed to Zn up to 1300–4000 mg kg^−1^ soil for 12 months under pot experiment. Similarly, Li et al. [28] observed a sharp decrease in AOB populations in a Cu-spiked (1200 mg kg^−1^) soil during a 21-day long microcosm study with grassland and cropland soils. In line with the potential impact on AOB population, the AOA/AOB ratio was clearly seen decreased in polluted soils while both AOA and AOB abundances were reduced significantly at DX site, where the Cu concentration was as high as 1333 mg kg^−1^. In a similar fashion, the *nirK*/*amoA* ratios observed in our study ranged from 0.74 to 7.92 (Table 3), indicating a greater abundance of denitrifiers (*nirK*) relative to ammonia oxidizers (AOA and AOB) in all of the soils except for the BGS at DBS site. Similarly, Hai et al. [45] reported the predominance of *nirK* gene relative to *amoA* genes (AOA and AOB) in the rhizosphere of sorghum in an agricultural soil.

N availability to microbes of soil, usually assessed with C/N ratio, could affect the N removal in soils. Here, N was generally higher in polluted soil than the background soil in both sites for less N consumed by rice with reduced production under metal pollution. However, C/N ratio seemed similar between the two sites both of polluted soils (14.4 and 14.8 respectively for DX and DBS) and of background soils (11.4 and 11.8 respectively for DX and DBS). The finding that, over BGS, a decrease in AOB in PS was higher in DBS than in DX but AOA-to-AOB ratio in PS decreased in DX and increased in DBS suggested a role of metal pollution situation rather than N concentration on the community abundance, This was in line with the finding of a recent study of the authors group [46] that N_2_O production was much higher (by∼350%) in metal polluted soil (C/N ratio 11.6) than by 190% in unpolluted soil (C/N ratio 11.8) when amended with polluted straw amendment.

### Changes in community structure of ammonia oxidizer and denitrifier in metal polluted soil

Based on DGGE analysis between BGS and PS, both AOB and AOA communities were distinguishable at DBS site with a higher pollution degree by multiple metals but Whereas, only AOA community at DX site with a low pollution degree but predominated by Cu. Phylogenetic analysis revealed that all the AOB sequences in our study belonged to the genus *Nitrosospira* (Fig. 2). The dominance of *Nitrosospira spp*. both in BGSs and PSs here was already reported in earlier studies [47], [48]. The clones from polluted soils in this study were affiliated with several clusters in the genus *Nitrosospira* as identified by Mertens et al. [24]. In the present work, sequences identified as belonging Cluster 4 were obtained only from the unpolluted soil at the DBS site, suggesting that multiple metal pollution of Cd, Pb, Cu and Zn had a strong impact on AOB community composition. However, it was unclear whether the metal tolerant populations in these heavy metal polluted soils were intrinsically tolerant to pollution or whether tolerance had been conferred by horizontal gene transfer. Some clones that were common to background soil were phylogenetically close to those that occurred in the polluted soil. As with the AOB community, the mechanistic details of AOA adaptations to heavy metal contamination remains unclear due to the limited ability to cultivate AOA species. Gene encoding AOA were also carried by members of the archaea that had been detected by molecular phylogenetic approaches, but that had not yet been cultured [49], [50].

The communities represented by *nirK* were distinguishable between BGS and PS soils at DX site, but not at DBS site. Here, phylogenetic analysis showed that the clones from the polluted and background soils at a single site could be sorted into different groups. Furthermore, the clones in the polluted soils were widespread among samples from both sites. A study by Prasad et al. [51] showed that heavy metal tolerance varied widely among bacterial genera. These include several lineages belonging to the *Proteobacteria* that appeared to be highly tolerant to metal contaminated environments [52], [53].

In addition to the influence of metal pollution, ammonia oxidizer and denitrifier community compositions were also affected by soil pH, SOC and TN. Soil organic carbon content is increased in polluted soils probably owing to inhibited decomposition rate [54]. On the other hand, soil pH had been found to affect microbial community structure directly by altering functional microbial groups [55], and indirectly by altering soil factors such as C and N substrate [56] and the availability of metals [57]. In this study, soil pH was lower but N higher in PS than in BGS at both sites, of course, both N and soil pH could be directly or indirectly affected by metal pollution [23]. There should be some interactions of soil chemical condition with metal pollution, for pH and organic matter could alter metal availability in soils to microorganisms.

### Relationship between community changes and potential nitrifying and denitrifying activities

Nitrification had been known sensitive to heavy metal pollution [58]–[60], with heavy metal addition often decreasing soil nitrification rates [26], [61]. Here, we observed that nitrifying activity was inhibited significantly in PSs compared to BGSs at both sites, which could be related to an altered soil microbial community composition with more resistant species. This could be partly explained by the remarkable decrease in both AOB and AOA populations with the metal pollution at both sites. AOB community composition was altered but its abundance unchanged in a long term Zn-polluted grassland, leading to an unchanged potential nitrification activity between polluted and unpolluted soils [62]. In contrast, a great decrease (up to 50%) in potential nitrification rates was found in copper spiked soils, with a simultaneous decrease in abundances of AOA and AOB plus a shift in AOB community structure and changes in the composition of the AOA community [63]. Of course, these community compositions could also subject to changes in soil pH and N level in soils under metal pollution [64], which could also be influenced with metal accumulation, as mentioned above. Nevertheless, metal impact on nitrification potential via metal toxicity or via alteration in abiotic conditions associated with C and N availability could be still a question.

Unlike nitrification activity, the potential denitrification activity was unchanged in the metal polluted soils at the DBS site with a soil pH of 5.5 but reduced significantly at DX site with Cu level up to 1333 mg kg^−1^ t and a low pH of 4.1. Apparently, this change was not related to difference in SOC and total N between the two sites. This seemed contrast to the reports that carbon additions lead to an increase in denitrifying activity but do not change the composition of the *nirK* community [65], [66]. An inhibition of denitrification activity had been widely recognized under highly elevated Cu level in soil though soil pH could be an important factor for denitrifying activity. A decrease in denitrification activity in soils spiked or polluted with metals including high Cu was reported for wetland soil [27] and for pasture soil [67]. Attard et al. [68] argued that a change in denitrifying activity could be associated with a change in denitrifier abundance but not with a change in denitrifier community structure under land use changes. Here, a several fold decrease in denitrification activity was seen in DX but not in DBS, which corresponded with a shift in the composition of the *nirK* community in PS from DX but not from DBS (section 4.2). Notably, changes in potential denitrification activity could not be predicted from changes in the abundance of functional gene targeted with the *nirK* primers. As shown by Heylen et al. [69] in a culture-independent study through the *nir* gene sequence analysis of cultivated denitrifier, functional *nir* gene diversity did not match well the denitrifier diversity. There were likely uncertainties regarding the *nirK* gene data. Nitrite reductase was considered the key enzyme in denitrification, containing either cytochrome cd1 encoded gene (*nirS* denitrifier) or copper encoded gene (*nirK* denitrifier), could catalyze the reduction of NO_2_^−^ to nitric oxide (NO). The *nirS* denitrifier appeared more abundant than *nirK* denitrifier, however, the latter could be more sensitive to soil environmental changes [70]. Recently, targeting both *nirK* and *nirS* genes in forest, grassland and agriculture systems had been proposed as an assay to elucidate the abundance and community structure of soil denitrifier [68], [70], [71]. However, it is still a question if this assay could better track the changes in denitrification activity with changes in denitrifying bacterial abundance in the polluted soils. Meanwhile, gene transcript numbers would be also a potential option to better predict the functional groups responsible for denitrification in these soils since they could reflect the active populations of the community.

## Conclusion

Significant changes both in the activities and community structure of ammonia oxidizers and denitrifier existed with metal pollution in an interaction with soil abiotic factors in rice paddies. A consistent decrease in the AOB abundance and nitrifying activity in polluted soil was observed in two sites studied. However, a sharp decrease in AOA abundance and denitrifying activity were seen only in highly Cu-polluted soil though lower pH and higher N was seen in polluted soil compared to the background soil in both sites. By using molecular techniques employing DGGE, we observed a shift in the community structure of AOA, and to a lesser extent, of AOB and denitrifier populations that were associated with different metal composition of the polluted soils. The pollution effects on microbial abundance differed between populations of *amoA* and *nirK* genes in a single site but these changes were not seen correlated to changes in nitrification or denitrification activities. This could suggest either a possible non-specific target of the primers conventionally used in soil study or complex interactions between soil properties and metal contents on the observed community and activity changes. This study suggested that metal pollution could exert impacts on soil microbial communities responsible for N transformation and thus on potential N_2_O production in rice paddies though the impacts on different communities could vary with metal composition and the associated changes in soil pH and N availability. However, future works would be required either with new molecular assays and/or on microbial responses to multiple metals under contrasting soil conditions in polluted agricultural soils.

## Supporting Information

Figure S1 **Principal component analysis (PCA) of DGGE profiles of AOB (a), AOA (b) and** ***nirK*** **(c) gene fragment from the soil samples at the two sites.** DX-B and DBS-B, background soil from site DX and DBS; DX-P and DBS-P, polluted soil from site DX and DBS. Similar symbols with same color in PCA plot indicate the replicate samples.
(TIFF)

Figure S2 **DGGE profiles of AOB (A), AOA (B) and** ***nirK*** **(C) gene fragment from the soil samples at the two sites.** M: 100 bp Marker. DX-B and DBS-B, background soil from site DX and DBS; DX-P and DBS-P, polluted soil from site DX and DBS. Arrows indicate the excised bands (B17–B27 and K14–K22) for sequencing.
(TIFF)

Table S1 Primer sets and thermal profiles used for the absolute quantification of functional target genes.
(DOCX)
