# Supplementary figures and images for: Abundance, Composition and Activity of Ammonia Oxidizer and Denitrifier Communities in Metal Polluted Rice Paddies from South China
Source: PLoS One. 2014 Jul 24;9(7):e102000. doi: 10.1371/journal.pone.0102000 (PMC4109924; doi:10.1371/journal.pone.0102000)

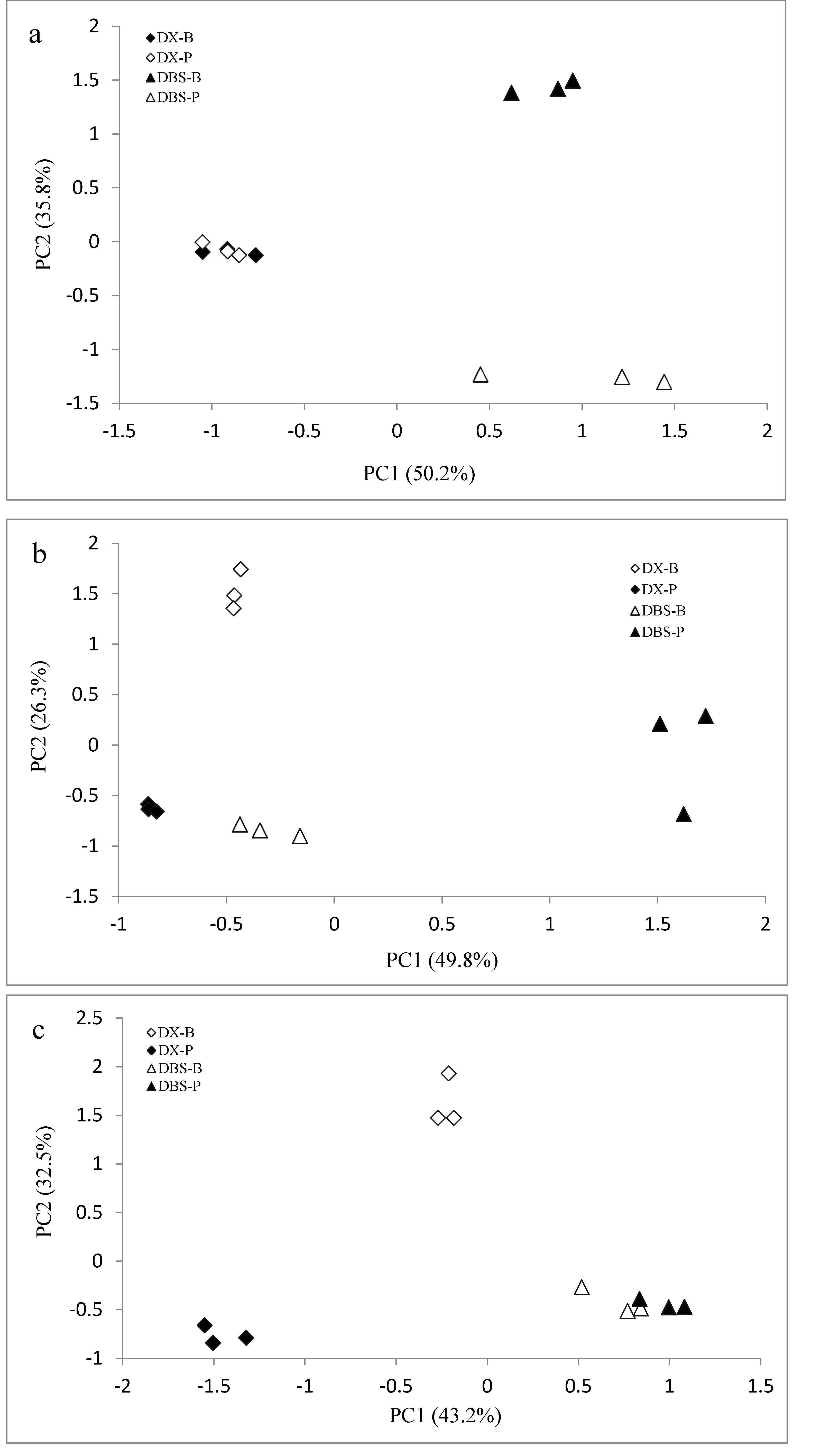

Supplement: Figure S1 — Principal component analysis (PCA) of DGGE profiles of AOB (a), AOA (b) and nirK (c) gene fragment from the soil samples at the two sites. DX-B and DBS-B, background soil from site DX and DBS; DX-P and DBS-P, polluted soil from site DX and DBS. Similar symbols with same color in PCA plot indicate the replicate samples. (TIFF) [file pone.0102000.s001.tiff]

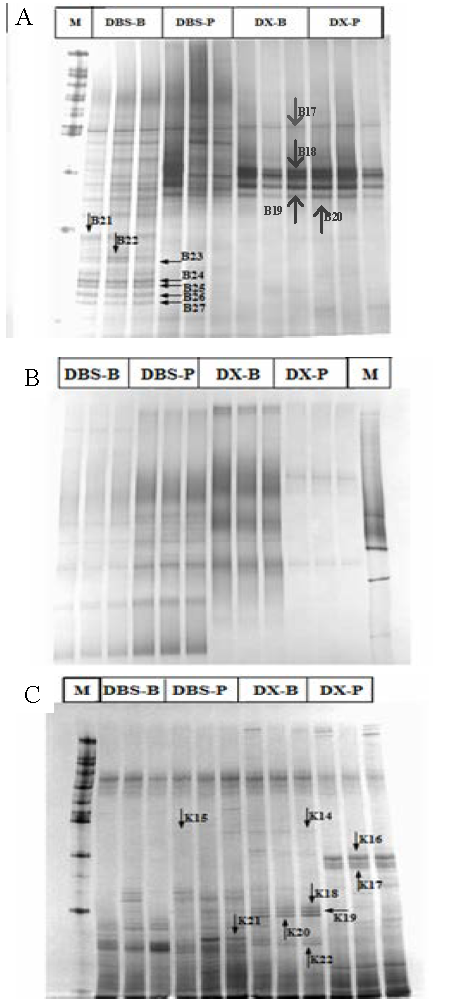

Supplement: Figure S2 — DGGE profiles of AOB (A), AOA (B) and nirK (C) gene fragment from the soil samples at the two sites. M: 100 bp Marker. DX-B and DBS-B, background soil from site DX and DBS; DX-P and DBS-P, polluted soil from site DX and DBS. Arrows indicate the excised bands (B17–B27 and K14–K22) for sequencing. (TIFF) [file pone.0102000.s002.tiff]
