# Supplementary material for: Abundance, Composition and Activity of Ammonia Oxidizer and Denitrifier Communities in Metal Polluted Rice Paddies from South China
Source: PLoS One. 2014 Jul 24;9(7):e102000. doi: 10.1371/journal.pone.0102000 (PMC4109924; doi:10.1371/journal.pone.0102000)
Supplement: Table S1 — Primer sets and thermal profiles used for the absolute quantification of functional target genes. (DOCX) [file pone.0102000.s003.docx]

**Table S1** Primer sets and thermal profiles used for the absolute quantification of functional target genes.

| Target gene | Primer set | Size | Thermal cycling profile | Reference |
| --- | --- | --- | --- | --- |
| *amoA* (AOB) | *amoA*-1F | 490bp | 95 ^◦^C (5 min); 40 cycles of 95 ^◦^C (1 min), 55 ^◦^C (1 min), and 72 ^◦^C (1 min). | McTavish et al. (1993) |
|  | *amoA*-2R |  |  |  |
| *aomA* (AOA) | Arch-*amoA*F | 635bp | 95 ^◦^C (5 min); 40 cycles of 95 ^◦^C (1 min), 55 ^◦^C (1 min), and 72 ^◦^C (1 min). | Francis et al. (2005) |
|  | Arch-*amoA*R |  |  |  |
| *nirK* | *nirK*876 | 165bp | 95 ^◦^C (5 min); 40 cycles of 94 ^◦^C (30 s), 58 ^◦^C (1 min), and 72 ^◦^C (1 min). | Henry et al. (2006) |
|  | *nirK*1040 |  |  |  |

AOB: ammonia oxidizing bacteria; AOA: ammonia oxidizing archaea.
